# Surgical repair of peripherally inserted central catheter extravasation via a transmanubrial approach

**DOI:** 10.1016/j.jvscit.2026.102160

**Published:** 2026-01-25

**Authors:** Masashi Bungo, Hisashi Uemura, Akifumi Nakamura, Taichi Sakaguchi

**Affiliations:** a Department of Cardiovascular Surgery, Hyogo Medical University, Nishinomiya, Hyogo, Japan; b Department of Thoracic Surgery, Hyogo Medical University, Nishinomiya, Hyogo, Japan

**Keywords:** Mediastinum, Abscess, Shoulder, Parenteral nutrition, Catheters

## Abstract

The transmanubrial approach (TMA) offers great exposure for managing complex superior mediastinal pathology while preserving functional anatomy. Here, we report a case of extravascular perforation caused by peripherally inserted central catheter with mediastinal abscess formation, which was successfully treated using a TMA. A 76-year-old woman developed a fever 3 months after the insertion of a peripherally inserted central catheter for parenteral nutrition. Imaging showed catheter tip migration with venous perforation, accompanied by abscess formation in the superior mediastinum. Owing to the anatomical difficulty in accessing this region, TMA was performed to remove the catheter and drain the abscess. The TMA provides optimal access while preserving neck and shoulder mobility, making it a suitable surgical option in older, frail patients.

Peripherally inserted central catheters (PICCs) are widely used as a well-tolerated and long-term venous access device; however, they may be associated with complications requiring urgent interventions. Commonly reported complications include deep vein thrombosis (30.6%),^1^ phlebitis (4% to 21%),^2^ catheter-related infections (3.0% to 5.7%),^2^ and late catheter tip migration (1.5%).^2^ Although rare, catheter tip migration may occur within days to months after insertion and may result in severe complications, including mediastinitis secondary to venous perforation. Surgical management poses a considerable challenge owing to limited exposure in the subclavian region. The transmanubrial approach (TMA) offers a promising solution by providing excellent anatomical exposure while preserving vital structures. Here, we present a case of vascular perforation and mediastinal abscess caused by PICC placement that was treated successfully using the TMA.

## Case report

A 76-year-old woman presented to a local hospital with dyspnea and fever and was admitted with a diagnosis of aspiration pneumonia. During hospitalization, a PICC was placed via the right upper arm for parenteral nutrition. The catheter functioned normally after placement. Although antibiotic therapy led to improvement of the pneumonia, her activities of daily living declined significantly during the hospital stay; thus, she was transferred to another facility.

Three months later, she developed a fever and elevated inflammatory markers and was readmitted to the previous hospital with suspected recurrent pneumonia. Computed tomography scan on admission revealed migration of the PICC tip inserted from the right upper arm, the presence of free air, and contrast enhancement in the superior mediastinum, raising suspicion of vascular perforation with abscess formation. She was subsequently referred to our hospital for PICC removal and surgical management of the mediastinal abscess.

**Fig 1:**
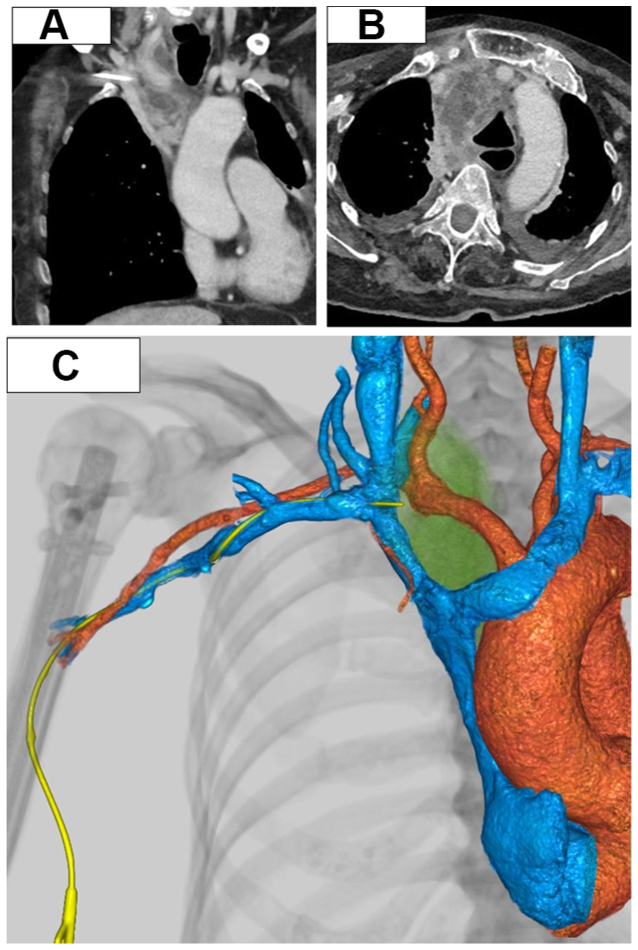
**(A)** Preoperative computed tomography image revealing extravascular perforation caused by PICC at the junction of the right brachiocephalic and subclavian veins. **(B)** Preoperative computed tomography image revealing abscess formation in the superior mediastinum. **(C)** Three-dimensional computed tomography image revealing extravasation of the PICC. *PICC*, peripherally inserted central catheters.

On admission to our institution, her vital signs were stable, but she was markedly emaciated and in poor general condition. The PICC (Bard Access Systems; double lumen, 5F) remained in place in the right upper arm. Laboratory findings revealed hypoalbuminemia (2.2 g/dL), no leukocytosis (white blood cell count of 5710/μL), and mildly elevated C-reactive protein (3.66 mg/dL). Contrast-enhanced computed tomography scan confirmed that the PICC, initially positioned in the superior vena cava, had migrated to the junction of the right brachiocephalic and subclavian veins, with associated mediastinal fluid collection and free air, leading to mediastinal abscess formation (Fig 1).

**Fig 2:**
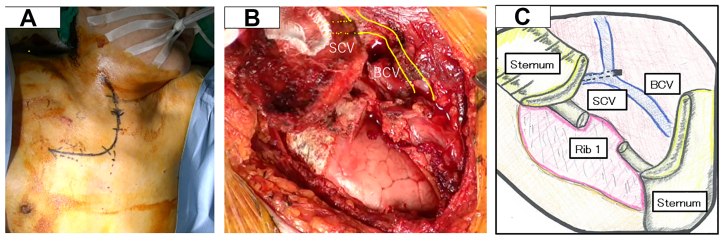
**(A)** Intraoperative view of the L-shaped skin incision for the TMA. **(B)** Intraoperative findings showing exposure of the right brachiocephalic vein (*BCV*) and subclavian vein (*SCV*). **(C)** Schematic illustration showing the right SCV flowing into the BCV at the surgical site of the transmanubrial approach (TMA).

Given the diagnosis of mediastinitis with abscess formation secondary to vascular perforation by the PICC, we planned surgical removal of the catheter and mediastinal drainage. Because of the anatomical complexity of the region, the TMA was selected. The procedure was initiated in the left lateral decubitus position with thoracoscopic access followed by repositioning to the supine position for the TMA. The right upper lobe of the lung was adherent to the parietal pleura and was dissected as extensively as possible. The anterior mediastinal pleura was markedly thickened and fibrotic owing to inflammation, and partial opening yielded turbid drainage. Thoracotomy was then performed via TMA. A 24F chest tube was placed through the thoracoscopic port toward the lung apex, and a 19F Blake drain (Ethicon) was placed in the anterior mediastinum. Only minimal debridement was required, and pulse lavage was not performed. The mediastinal tissue was dissected, the anterior mediastinum was drained, and the right brachiocephalic and subclavian veins were exposed. Although the PICC tip was not directly visualized, localized bleeding was noted on the venous wall. The PICC was removed through the original insertion site under direct visualization, and the suspected perforation site was repaired with direct suturing (Fig 2).

The operative time was 2 hours and 45 minutes, with an estimated blood loss of 240 mL. Cultures of the mediastinal drainage were negative. Postoperative antibiotics were continued until postoperative day 20. Both the chest tube and mediastinal drain were removed on postoperative day 7. The postoperative course was uneventful, and the patient was transferred to a rehabilitation facility on postoperative day 58. The patient provided written informed consent for publication.

## Discussion

Surgical access to the subclavian vessels is challenging owing to their deep location beneath the sternum and clavicle, as well as the anatomical complexity of this region. The presence of the clavicle and ribs further restrict the operative field and impairs visualization; furthermore, conventional supraclavicular and infraclavicular incisions often provide inadequate exposure. Consequently, more invasive approaches, such as median sternotomy or clavicular resection, are required, although these carry risks of postoperative functional impairment and cosmetic complications. Endovascular therapy has emerged as a less invasive option for treating subclavian vessel injuries.^3^, ^4^, ^5^ However, its suitability depends on the underlying pathology and anatomical features. In certain cases, endovascular devices may be inappropriate owing to the wide range of shoulder motion or the risk of device kinking at the level of the first rib.

TMA, first described by Grunenwald and Spaggiari^6^ in 1997 as an access route for superior thoracic lesions, preserves the sternoclavicular joint while providing excellent exposure of the subclavian region. It has since been applied to the treatment of superior mediastinal and apical lung tumors^6^ and subclavian artery aneurysms.^7^ Moreover, its safety and efficacy have been reported in diverse patient populations, including pediatric patients,^8^ and its versatility is further demonstrated by reports of its successful removal of iatrogenically misplaced catheters.^9^

Although TMA requires partial resection of the superolateral manubrium, it does not result in functional or cosmetic hindrances. The clavicle is preserved and most of the pectoralis major muscle remains intact, thereby maintaining postoperative shoulder girdle mobility. The incidence of wound-related complications has been reported to be low.^6^, ^7^, ^8^ Owing to these functional and cosmetic benefits, TMA has also been applied successfully in pediatric patients, with no postoperative thoracic deformities, and localized atrophy of the pectoralis major being the only long-term complication observed in a small subset of patients.^8^

In the present case, TMA facilitated safe vascular repair and thorough debridement of the mediastinal abscess without compromising shoulder or cervical mobility or cosmetic outcomes. These aspects are particularly critical in frail patients with reduced physiological reserve. Furthermore, the combined use of thoracoscopic drainage for the mediastinal abscess may have contributed to lower the risk of residual infection and prevention of postoperative osteomyelitis.

## Conclusions

TMA is a simple and safe surgical technique with wide applicability across diverse pathological and clinical contexts. The present case highlights its utility in managing complex situations involving vascular injury with concomitant infection. TMA provides excellent surgical exposure of the entire subclavian vasculature, preserves shoulder joint function, and helps to prevent wound-related complications, even in emergency settings. Although open surgery remains the principal method for most cases requiring subclavian vessel repair, TMA should be considered a valuable surgical alternative in such scenarios.

## Funding

None.

## Disclosures

None.
